# Understanding Prospective Physicians’ Intention to Use Artificial Intelligence in Their Future Medical Practice: Configurational Analysis

**DOI:** 10.2196/45631

**Published:** 2023-03-22

**Authors:** Gerit Wagner, Louis Raymond, Guy Paré

**Affiliations:** 1 Faculty Information Systems and Applied Computer Sciences University of Bamberg Bamberg Germany; 2 Université du Québec à Trois-Rivières Trois-Rivières, QC Canada; 3 Department of Information Technologies, École des Hautes Études commerciales Montréal Montréal, QC Canada

**Keywords:** artificial intelligence, medical education, attitudes and beliefs, knowledge and experience, behavioral intentions, fuzzy-set qualitative comparative analysis, fsQCA

## Abstract

**Background:**

Prospective physicians are expected to find artificial intelligence (AI) to be a key technology in their future practice. This transformative change has caught the attention of scientists, educators, and policy makers alike, with substantive efforts dedicated to the selection and delivery of AI topics and competencies in the medical curriculum. Less is known about the behavioral perspective or the necessary and sufficient preconditions for medical students’ intention to use AI in the first place.

**Objective:**

Our study focused on medical students’ knowledge, experience, attitude, and beliefs related to AI and aimed to understand whether they are necessary conditions and form sufficient configurations of conditions associated with behavioral intentions to use AI in their future medical practice.

**Methods:**

We administered a 2-staged questionnaire operationalizing the variables of interest (ie, knowledge, experience, attitude, and beliefs related to AI, as well as intention to use AI) and recorded 184 responses at *t*_0_ (February 2020, before the COVID-19 pandemic) and 138 responses at *t*_1_ (January 2021, during the COVID-19 pandemic). Following established guidelines, we applied necessary condition analysis and fuzzy-set qualitative comparative analysis to analyze the data.

**Results:**

Findings from the fuzzy-set qualitative comparative analysis show that the intention to use AI is only observed when students have a strong belief in the role of AI (individually necessary condition); certain AI profiles, that is, combinations of knowledge and experience, attitudes and beliefs, and academic level and gender, are always associated with high intentions to use AI (equifinal and sufficient configurations); and profiles associated with nonhigh intentions cannot be inferred from profiles associated with high intentions (causal asymmetry).

**Conclusions:**

Our work contributes to prior knowledge by showing that a strong belief in the role of AI in the future of medical professions is a necessary condition for behavioral intentions to use AI. Moreover, we suggest that the preparation of medical students should go beyond teaching AI competencies and that educators need to account for the different AI profiles associated with high or nonhigh intentions to adopt AI.

## Introduction

### Background

Artificial intelligence (AI), which is broadly defined as the use of a computer to model intelligent behavior with minimal human intervention [1], has the potential to transform or even revolutionize medicine [2]. In his seminal book, entitled “Deep Medicine: How Artificial Intelligence Can Make Health Care Human Again,” Topol [3] highlighted AI’s potential to improve the lives of physicians and patients. The promise of clinical AI algorithms ranges from image-based diagnosis in radiology, ophthalmology, and dermatology [4-6] to patient monitoring in cardiology and endocrinology [7,8] to the prediction of cardiovascular and kidney diseases [9,10], to name a few. In these areas, AI could offer valuable diagnostic and predictive insights concerning subtle changes to cue prospective physicians to initiate preventive measures as well as timely and accurate interventions [2,11].

For the potential benefits associated with AI use to materialize to their full potential, both current and future generations of physicians must be able to navigate with ease in an ever-changing digital environment. Accordingly, a growing academic literature has emerged on the attitudes of physicians toward AI, most of which concerns radiologists. According to these studies, the perception of AI among this group of specialists ranged between acceptance with enthusiasm and skepticism owing to the fear of being displaced by the technology [12,13]. Other surveys concerned all physicians, irrespective of their specialty. For instance, Oh et al [14] surveyed 669 physicians practicing in South Korea. Although most respondents considered AI useful in medical practice, only 5.9% (40/669) said that they had good familiarity with this technology. The ability to analyze vast amounts of high-quality, clinically relevant data in real time was seen as the main advantage of using AI, and a vast majority of the respondents (558/669, 83.4%) agreed that the area of medicine in which AI would be the most useful is disease diagnosis.

More recently, Scheetz et al [15] conducted a web-based survey of 632 fellows and trainees of 3 specialties (ophthalmology, radiology or radiation oncology, and dermatology) in Australia and New Zealand. Findings revealed that 71% (449/632) of the respondents believed that AI would improve their field of medicine, and 85.7% (542/632) felt that medical workforce needs would be affected by AI within the next decade. Improved disease screening and streamlining of monotonous tasks were identified as key benefits of AI. Finally, Paré et al (Paré, G, unpublished data, March 2022) investigated the assimilation of digital health technologies by Canadian family physicians to further understand the breadth and depth of their use in clinical practice for the diagnosis, treatment, and prevention of diseases and for the monitoring of chronic patients. A slight majority (422/768, 54.9%) of the respondents indicated that they were open to using AI for medical diagnosis purposes.

Although education has been identified as a priority to prepare future physicians for the successful implementation of AI in health care [15,16], to our knowledge, only a few studies have investigated medical students’ attitudes toward AI and their beliefs concerning the relevance of introducing AI-related material as a standard part of the curriculum. For instance, Sit et al [17] explored the attitudes of 484 United Kingdom medical students regarding training in AI technologies, their understanding of AI, and career intention toward radiology. Findings revealed that medical students do not feel adequately prepared to work alongside AI but understand the increasing importance of AI in health care and would like to receive formal training on the subject. Another example is the study by Park et al [18] that surveyed 156 radiology students in the United States. Over 75% (117/156) of the students agreed that AI would play a major role in the future of medicine, and 66% (103/156) of the students believed that diagnostic radiology would be the specialty most greatly affected by AI. Approximately half of the students (69/156, 44.2%) reported that AI made them less enthusiastic about radiology as a medical specialty.

In light of the aforementioned information, little empirical knowledge is available on medical students’ views on, familiarity with, and intention to use AI-based health technologies (AIHTs), including big data analytics and machine learning–based applications that are promised to have profound medical and societal impacts (eg, the study by Galetsi et al [19]). Further, prior studies mainly surveyed radiology students (eg, the study by Park et al [18]) or focused on students’ intention to use a specific AI-based application (eg, the study by Tran et al [20]). Importantly, prior surveys soliciting medical students’ opinions were conducted before the COVID-19 pandemic and are highly descriptive and atheoretical in nature. This study aims to fill these gaps. More precisely, we adopt a *configurational* perspective [21] to investigate the AI profiles of prospective physicians, that is, to identify the different configurations of factors that characterize these individuals with regard to AI. In addition, this study aimed to identify the AI profiles that are associated with a strong intention on the part of prospective physicians to use AIHTs in their future medical practice.

As explained in the *Theoretical Foundations* section, the configurational approach is based on the premise that there are specific combinations of prospective physicians’ AI knowledge, experience, attitudes, and beliefs that positively influence their intention to use AIHTs in medical practice [22]. Therefore, the first research question answered by this study is the following: *In a medical school context, what are the different AI profiles of prospective physicians that are associated with a strong intention on their part to use AIHTs in their future medical practice?* Additionally, given that the configurational approach allows for causal asymmetry, the second question is as follows: *What are the different AI profiles that do not allow these individuals to have a strong intention to use AIHTs in their future practice?*

### Theoretical Foundations

**Figure 1:**
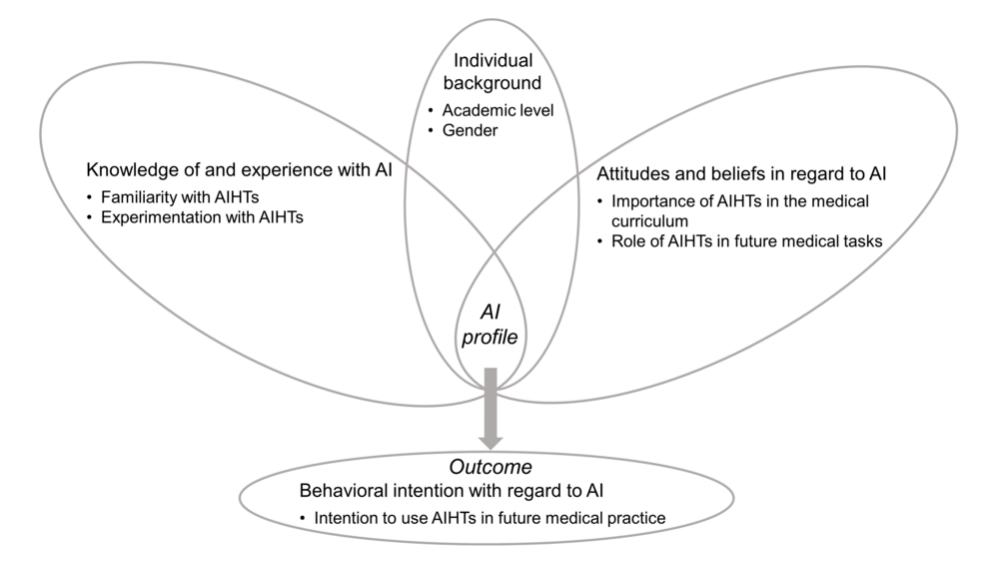
Configurational model of prospective physicians’ behavioral intention with regard to artificial intelligence (AI). AIHT: AI-based health technology.

The configurational model of prospective physicians’ behavioral intention with regard to AI, empirically investigated in this study, is presented in Figure 1. This model first assumes that the AI profiles of prospective physicians are made up of two main components: (1) knowledge of and experience with AI and (2) attitudes and beliefs with regard to AI. Our model also assumes that different AI profiles will be associated with different levels of behavioral intention with regard to AI. This assumption is based on the basic tenet of configurational theory, which follows the *systems* (rather than *variance*) approach [23] and seeks to further explain complex societal, organizational, group, and individual phenomena by identifying synergistic combinations of interacting causal conditions [21].

The first component of our configurational model refers to prospective physicians’ familiarity and experimentation with AIHTs. In the present context, familiarity with AIHTs is mainly within a medical student’s own control (endogenous factor). It is closely related to the concept of computer self-efficacy [24], which is included in many IT adoption models. For its part, experimentation with AIHTs is largely influenced by external factors (exogenous factor). It is associated with the concept of *facilitating conditions* included in the technology acceptance model (TAM), a theory that models how potential users come to adopt a new technology [25]. Facilitating conditions are external factors that influence an individual’s perceptions of the difficulty with which a task (eg, use of AIHTs) may be performed [26]. In medical teaching, facilitating conditions such as digital skills training would thus enhance students’ assimilation of instructional technologies [27]. In this study, facilitating conditions are operationalized as medical students’ level of hands-on experimentation with AI-based tools during their medical education.

Next, the second configurational component refers to prospective physicians’ attitudes and beliefs with regard to AI. According to Triandis’ [28] theory of interpersonal behavior, individuals’ behavioral intention is influenced by their attitudes and beliefs with regard to the behavior. On the one hand, attitudes toward technology use have mainly been conceptualized through the “perceived usefulness” component of the TAM, defined as the degree to which individuals deem that using a particular technology would enhance their work performance [25]. Adapted to this study’s context, perceived usefulness refers to prospective physicians’ perceptions of the greater importance that should be afforded to AIHT training within their medical studies. On the other hand, beliefs concerning technology use have mainly been conceptualized through the “perceived consequences” of such use [28], that is, through individuals’ perceptions of the value expected from the intended behavior [29]. In this study, we assessed medical students’ belief in the role that AIHTs are expected to play in support of their future medical tasks such as the prevention and diagnosis of illnesses.

Following prior research on digital health training (eg, the study by Vossen et al [30]) as well as various studies testing the TAM (eg, the study Venkatesh [31]), 2 individual factors were included, namely, gender and academic level, as individual background variables to add a contextual component to our configurational model. Here, we simply assume that these factors are likely to be associated with the prospective physicians’ AI profile, which, in turn, will be associated with their behavioral intention with regard to AI.

Whereas the theoretical background of our study is constituted by the previously mentioned configurational theory and by behavioral theories such as Triandis’ [28] theory of interpersonal behavior and the TAM, the theoretical foreground is founded upon the theorization of the task-technology fit concept. This last theory’s basic tenet is that a technology will be adopted to the extent that it is perceived to be well suited to the work tasks of the individuals whom it is meant to support, that is, suited to their tasks’ complexity, uncertainty, interdependence, and autonomy [32]. In our case, the notion of *fit* implies an understanding of how best to match AI-based tools with specific medical tasks (eg, diagnosing an illness) in specific medical contexts (eg, in emergency care) [33]. This led us to propose that the prospective physicians’ intention to use AIHTs in their future practice would primarily depend on the perceived consequences of such use, that is, on the prospective physicians’ belief that using AI-based tools will render them more effective in accomplishing their medical tasks. In turn, we also assume that such beliefs would be primarily conditioned by the prospective physicians’ evolving knowledge of and experience with AIHTs and by their concomitantly evolving attitude toward the AI training received during their medical studies [34].

## Methods

### Overview

This study was conducted at the University of Montréal’s medical school in Canada. During the 5-year long undergraduate medical curriculum, no formal digital health education or training is provided to students. However, students have access to the EDUlib web-based training platform that offers educational content on a variety of subjects, including health and information technologies, as well as to symposia and conferences on different aspects of digital health. The study population consisted of 1367 medical students from the University of Montréal. The survey questionnaire was administered in 2 phases: an initial survey (*t*_0_) in February 2020, before the COVID-19 pandemic, and a replication survey (*t*_1_) in January 2021, during the pandemic.

As we were unable to locate any preexisting questionnaire that assessed the variables included in our research, we developed our own measurement instrument. The items broadly align with those used in related contexts (eg, the study by Zigurs and Khazanchi [33]). The survey design underwent several rounds of iteration, and final validation was performed with a group of 10 medical students from the University of Montréal who were excluded from the sampling population.

The measurement of the research variables was based on the abovementioned literature on medical education in AI-enabled digital health technologies. The “experimentation with AIHTs,” “familiarity with AIHTs,” and “importance of AIHTs in the medical curriculum” variables were each measured with three 5-point scales (AI, machine learning, and big data analytics). The “role of AIHTs in future medical tasks” variable was measured with five 5-point scales pertaining to the potential effect of AIHT on medical tasks (prevention, diagnosis, treatment, prognosis, and patient-physician relationship). The outcome variable, “intention to use AIHT in future medical practice,” was measured through the summation of 8 dichotomous scales (yes or no) pertaining to the use of AIHTs in support of medical activities (radiological image analysis, photographical image analysis, pathological image analysis, diagnosis, prognosis, therapeutic planning, patient history data analysis, evaluation, and the monitoring of patient-physician communication). The full measurement instrument is presented in Multimedia Appendix 1.

To analyze the AI profiles associated with high or nonhigh intentions to use AIHTs, we performed fuzzy-set qualitative comparative analyses (fsQCAs) [34,35] in combination with analyses of necessity [36]. In a nutshell, fsQCA is a second-generation configurational analysis method that uses Boolean algebra for determining different configurations of elements that generate the same outcome [37]. In this method, each configurational element (or causal condition) is considered a *fuzzy set*. Consistent with the configurational theory, fsQCA allows for equifinality and causal asymmetry [22]. Specifically, in explaining prospective physicians’ behavioral intention toward AI adoption, the configurational approach allows us to account for complex and nonlinear relationships among the knowledge, experience, attitudes, and beliefs of these individuals with regard to AI as well as to account for *equifinality*. In this study, equifinality is the possibility for prospective physicians to have an equally strong intention to use AIHTs while showing different AI profiles, that is, through different configurations of conditions that *cause* the intention [38]. In other words, equifinality allows configurational elements (ie, the elements forming the prospective physicians’ AI profiles) to be combined in multiple ways to equally produce the outcome of interest (ie, a high level of behavioral intention), which means that the same element might be present in one *high-intention* AI profile but might be absent in another. Thus, the same configurational element (or causal condition), for example, a high level of familiarity with AIHTs, could be associated with high intention in one profile but not in other profiles, in which the prospective physicians’ intentions depend on how the familiarity is configured with the other elements that form the AI profile. This approach also allows for *causal asymmetry*, that is, the possibility that the AI profiles associated with the presence of a strong intention to use AIHTs differ from the profiles associated with the absence of such an intention [22].

In line with the methodological guidelines for fsQCA [39,40], we completed the steps of calibration, necessity analysis, truth table construction, and sufficiency analysis, as explained in the *Results* section. The fsQCA was conducted with the QCA (version 3.0) software [41].

### Ethics Approval

The survey questionnaire was approved by the ethics committee at the University of Montréal on October 29, 2019 (#CERSES-19-108-D). Informed consent was obtained from all participants. All methods were executed in accordance with relevant guidelines and regulations.

## Results

### Overview

Of the 1367 students, 184 (13.46%) students responded to the initial survey at *t*_0_, whereas 138 (10.1%) responded to the replication survey at *t*_1_. As shown in Table 1, most participants were women (119/184, 64.7% at *t*_0_ and 96/138, 69.6% at *t*_1_), and the number of participants in their third year or later of medical training (108/184, 58.7% at *t*_0_ and 78/138, 56.5% at *t*_1_) was more than the number of participants in their first or second year.

**Table 1:** Profile of the respondents.

Medical students’ background		*t*_0_ (n=184), n (%)	*t*_1_ (n=138), n (%)
**Academic level**			
	Preparatory year (year 1)	40 (21.7)	28 (20.3)
	First year preclinical (year 2)	36 (19.6)	32 (23.2)
	Second year preclinical (year 3)	43 (23.4)	56 (40.6)
	First year internship (year 4)	33 (17.9)	8 (5.8)
	Second year internship (year 5)	32 (17.4)	14 (10.1)
**Gender**			
	Women	119 (64.7)	96 (69.6)
	Men	65 (35.3)	42 (30.4)
Age (years), mean (SD; range)		22.9 (3.5; 18-38)	22.6 (2.7; 18-35)

The reliability and descriptive statistics of the research variables for the 2 samples (*t*_0_ and *t*_1_) are presented in Table S1 of Multimedia Appendix 2. Note that, overall, the sampled prospective physicians showed rather low levels of knowledge of AIHTs and experience with AIHTs. When comparing the variable means between the *t*_0_ and *t*_1_samples, a significant difference (*P*=.047) was found for a single variable, indicating that the prospective physicians at *t*_1_ (peri–COVID-19 pandemic) were less familiar with AIHTs, albeit slightly, than those at *t*_0_ (pre–COVID-19 pandemic). Overall, these 2 samples thus appeared to be quite similar, notwithstanding the advent of the COVID-19 pandemic after the initial survey. The correlation matrices of the research variables (*t*_0_ and *t*_1_) are presented in Table S2 of Multimedia Appendix 2.

With respect to the measurement properties of the research variables, one must first note that our measure of the outcome variable, intention to use AIHTs, is of the “index” rather than “scale” type. In contrast to scale measures, index measures tend to follow a Poisson type rather than a normal distribution and regroup elements not expected to be highly intercorrelated, hence the inappropriateness of using the Cronbach α coefficient to assess the internal consistency of such measures [42]. As shown in Table S1 in Multimedia Appendix 2, all α coefficients above the 0.80 threshold confirm the internal consistency of the 4 scale measures, and the average extracted variance of these measures confirm their convergent validity (average extracted variance>0.50).

Next, we examined the correlation matrix of the 4 scale variables to ascertain whether any 2 of these correlated above the 0.71 threshold, as this would indicate a strong risk of common method bias (CMB) in our data [43] and a lack of discriminant validity [44]. As shown in Table S2 in Multimedia Appendix 2, this was not the case. The “marker variable” CMB detection technique was also called upon [45]. The recommended procedure for applying this technique post hoc was used; that is, the smallest correlation among the scale variables (0.08 at *t*_0_ and 0.06 at *t*_1_) was used as a reliable estimate of common method variance (CMV) to calculate CMV-adjusted correlations [46]. Given that many of these adjusted correlations (33% at *t*_0_ and 66% at *t*_1_) were nonsignificant (*P*˃.05) and that the originally significant correlations among the variables remained significant when adjusted for CMV [47], it further appeared that CMB was not a major threat in this study.

Consistent with the configurational theory [21] and as opposed to covariance-based or component-based structural equation modeling techniques such as partial least squares regression, the configurational analysis method implemented in fsQCA assumes complex, nonlinear causality [22] and allows for equifinality and causal asymmetry [48]. The principal contribution of fsQCA lies in its ability to evaluate the relation between a configuration of elements and an outcome. The analysis of our configurational model was preceded by a direct fuzzy-set *calibration* of 5 of the 7 research variables, as it is recommended when Likert-type scales and indexes are used for variable measurement [48]. For each of our research variables, we thus identified the 3 points of fuzzy-set membership (fully-in, crossover, and fully-out) using percentiles, as recommended in the fsQCA literature [49]. For their part, the individual background variables—academic level and gender, measured as binary variables—constituted “crisp” sets (fully-in=1 and fully-out=0).

Although we first described fsQCA with regard to the relationship between the desired outcome and the case sets built for each causal condition (or configurational element), the main advantage of this technique lies in its capacity to analyze relationships between configurations (ie, combinations of causal conditions) and the outcome [37]. As the configurations are built through Boolean addition of individual causal conditions, a condition’s fuzzy-set score indicates its degree of membership in the solution.

The fsQCA technique starts its configurational analysis by creating a truth table of 2*k* rows, where each row represents a possible configuration combining *k* individual causal conditions [50]. This table is sorted on the basis of the frequency and consistency, where frequency represents the number of observations for each possible configuration, and consistency estimates “the degree to which cases correspond to the set-theoretic relationships expressed in a solution” [22]. Given our large-sized sample, we set the frequency threshold at 3; hence, all configurations with a frequency of ≤2 were deleted from further analysis. Furthermore, we applied the recommended threshold of 0.80 for consistency [51], which is also the default value in the fsQCA version 3.0 software used in this study. Hence, for configurations below the consistency threshold, the outcome variable was set at 0 and for the rest at 1, given that these configurations are the ones that fully explain the outcome [50].

### Configurational Analysis (*t*_0_)

#### Overview

The first step in fsQCA is the analysis of the configurational elements that are deemed *necessary* for the outcome (Table 2). Generally, the necessity of a causal condition is assessed by its consistency, that is, by the extent to which members in this condition (eg, prospective physicians believing the role of AIHTs in their future medical tasks to be highly important) show membership in the outcome (eg, prospective physicians having a high intention to use AIHTs in the future). Within fsQCA, a causal condition is deemed to be necessary for an outcome when its consistency score exceeds the threshold of 0.90 [37]. However, necessary condition analysis (NCA) provides a more suitable approach, especially for the necessity analyses of fuzzy-set conditions (derived from continuous variables). NCA is better suited for our data set because it is more aligned with in-degree necessary conditions, relying on ceiling line calculations that are more flexible than the dichotomous bisection underlying fsQCA necessity analyses [49]. The NCA analyses reported in Multimedia Appendix 3 suggest that prospective physicians’ strong beliefs in the role of AIHTs in their future medical tasks is a necessary condition for behavioral intentions. This finding is also corroborated by the occurrence of the same condition across all high-intention configurations, which is considered indicative of a necessary condition in fsQCA approaches [49].

**Table 2:** Analysis of the necessary configurational elements (*t*_0_)

Configurational element		High intention^a^ (to use AIHTs^b^ in future practice)			Nonhigh intention^c^ (to use AIHTs in future practice)	
		Consistency	Coverage	Consistency		Coverage
**Knowledge of and experience with AI^a,d^**						
	Familiarity with AIHTs	0.023	0.983	0.999		0.450
	Experimentation with AIHTs	0.447	0.679	0.736		0.516
**Attitudes and beliefs with regard to AI^a^**						
	Importance of AIHTs in the medical curriculum	0.736	0.705	0.615		0.651
	Role of AIHTs in future medical tasks	0.801	0.887	0.873		0.779
**Individual background^e^**						
	Academic level	0.584	0.553	0.410		0.441
	Gender	0.620	0.532	0.320		0.402

^a^Calibration: fully-in=top quartile, crossover=median, and fully-out=bottom quartile.

^b^AIHT: artificial intelligence–based health technology.

^c^Negated set (~).

^d^AI: artificial intelligence.

^e^Crisp set: fully-in=1 and fully-out=0.

The next step in fsQCA allows one to analyze the configurational elements that, together, are *sufficient* to produce the chosen outcome [37]. That is, using Boolean algebra and counterfactual analysis, fsQCA effectuates a logical reduction of the truth table into 3 types of solutions that combine the causal conditions that are deemed sufficient to achieve the desired outcome: parsimonious solutions, intermediate solutions, and complex solutions. Owing to its difficult interpretation and poor applicability, the complex solution—which produces all possible configurations of conditions—is simplified into the parsimonious and intermediate solutions. The intermediate solution is obtained through a counterfactual analysis of the complex and parsimonious solutions. The parsimonious solution yields the “core” conditions, whereas the “peripheral” conditions are those that are included in the intermediate solution but not in the parsimonious solution [37]. Therefore, the “core” conditions are those found to strongly influence the outcome and cannot be left out from any configuration, whereas the “peripheral” conditions have lesser influence on the outcome and, therefore, may be exchangeable (with other peripheral conditions) or even expendable. For interpreting results, it is recommended to combine the parsimonious and intermediate solutions to identify the core and peripheral conditions in the resulting configurations [22]. Now, the peripheral conditions may be regarded as “complementary” or “contributing” configurational elements in that they make sense as important causal conditions; they may thus be removed from a configuration only if one is willing to make assumptions that run counter to the existing theoretical and substantive knowledge [37].

#### Configurations for High Intentions to Use AIHTs in Future Medical Practice (*t*_0_)

**Figure 2:**
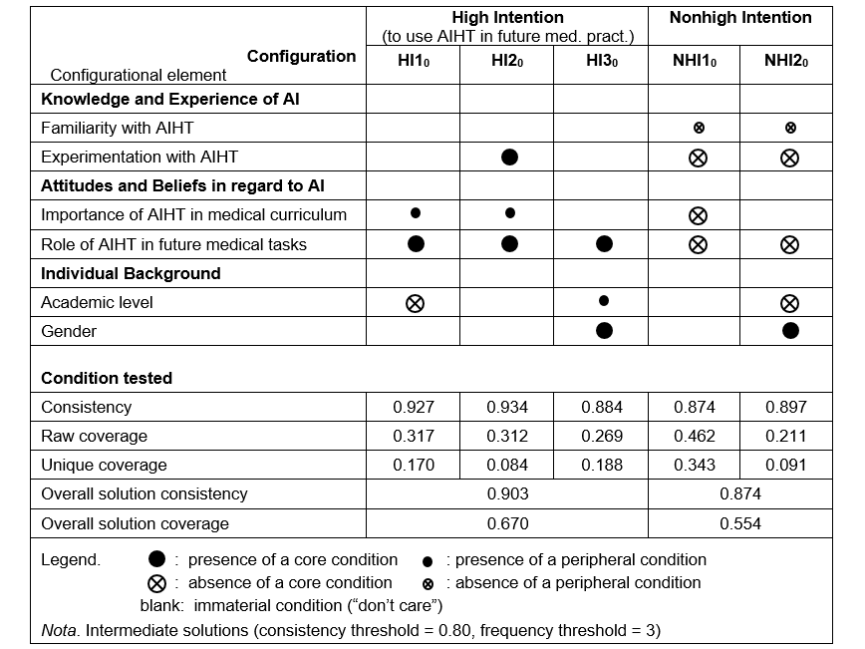
Configurations for the presence and absence of a high intention to use artificial intelligence (AI)–based health technologies (AIHTs) in future medical practice (*t*_0_). HI: high intention; med.: medical; NHI: nonhigh intention.

In demonstrating equifinality, the results of the fsQCA-based sufficiency analysis identify 3 intermediate solutions, that is, 3 causal configurations equally associated with a high intention to use AIHTs in future medical practice (*HI*1_0_, *HI*2_0_, and *HI*3_0_). The overall solution coverage indicates the proportion of cases that are covered by all reported configurations, whereas the overall solution consistency assesses the degree to which the configurations are subsets of the outcome. Note that, as shown in Figure 2, we use the notation introduced by Ragin [37]: black circles represent the presence of a condition, circles with a cross-out indicate the absence of the condition, large circles represent core conditions, small circles represent peripheral conditions, and blank spaces represent an immaterial condition (or a situation characterized by a “don’t care” in which one condition may be either present or absent without altering the outcome). The 3 intermediate solutions derived from fsQCA appear as follows in Figure 2:

- The first high-intention configuration, *HI*1_0_, highlights the need for prospective physicians to have a strong belief in the role of AIHTs in supporting their future medical tasks (core condition) and, secondarily, to have a favorable attitude toward the importance of AI in the medical curriculum (peripheral condition). Furthermore, *HI*1_0_ is under the (core) condition that these individuals be in their first or second year of medical education.
- The second configuration, *HI*2_0_, also highlights the need to have a strong belief in the role of AIHTs in future medical tasks (core condition) and, secondarily, a favorable attitude toward the importance of AI in the medical curriculum (peripheral condition). However, *HI*2_0_ also includes a sufficient level of experimentation with AIHTs as a (core) condition for prospective physicians to have a strong intention to use AIHTs in their future practice, irrespective of their academic level and gender.
- The last configuration, *HI*3_0_, is the most parsimonious, in that it only includes (as a core condition) having a strong belief in the role of AIHTs in future medical tasks under the added condition that the prospective physician be a woman (core condition) and that they be in their third or later year of medical education (peripheral condition).

Thus, at *t*_0_, there appears to be 3 different ways (or *causal recipes*) for prospective physicians to develop a strong intention to eventually use AIHTs in their future medical practice.

#### Configurations for Nonhigh Intention to Use AIHTs in Future Medical Practice (*t*_0_)

In addition to equifinality, the configurational approach taken here allows for causal asymmetry, that is, the possibility that the causal conditions for the presence of the preferred outcome will differ from those for its absence [22]. As this approach allows for nonlinearity in causation, the same configurational element may have different causal roles within different configurations. In demonstrating causal asymmetry (Figure 2), further results of the fsQCA analysis identify 2 causal configurations associated with nonhigh intention to use AIHTs in medical practice (*NHI*1_0_ and *NHI*2_0_), that is, with the absence—rather than the presence—of a strong intention on the part of prospective physicians. Here, the absence of a strong belief in the role of AIHTs in prospective physicians’ future medical tasks is the core condition that is shared by both non–high-intention configurations, thus reinforcing the necessity of this last configurational element. However, asymmetry is observed because the lack of experimentation with AIHTs is also a core condition that is shared by the 2 configurations. These last 2 core conditions may thus be considered as necessarily “preventing” prospective physicians from having a strong intention to use AIHTs in their future practice.

### Configurational Analysis (*t*_1_)

#### Overview

Similar to the results of the necessity analysis of the *t*_0_ data and as presented in Table 3, results of such an analysis of the *t*_1_ data indicate that no configurational element appears to be individually necessary for prospective physicians to have a high intention to use AIHTs.

**Table 3:** Analysis of the necessary configurational elements (*t*_1_).

Configurational element		High intention^a^ (to use AIHTs^b^ in future practice)			Nonhigh intention^c^ (to use AIHTs in future practice)	
		Consistency	Coverage	Consistency		Coverage
**Knowledge of and experience with AI^a,d^**						
	Familiarity with AIHTs	0.638	0.614	0.590		0.615
	Experimentation with AIHTs	0.327	0.612	0.788		0.534
**Attitudes and beliefs with regard to AI**						
	Importance of AIHTs in the medical Curriculum	0.758	0.662	0.605		0.710
	Role of AIHTs in future medical tasks	0.851	0.863	0.862		0.849
**Individual background^e^**						
	Academic level	0.566	0.506	0.436		0.496
	Gender	0.691	0.503	0.301		0.489

^a^Calibration: fully-in=top quartile, crossover=median, and fully-out=bottom quartile.

^b^AIHT: artificial intelligence–based health technology.

^c^Negated set (~).

^d^AI: artificial intelligence.

^e^Crisp set: fully-in=1 and fully-out=0.

#### Configurations for High Intention to Use AIHTs in Future Medical Practice (*t*_1_)

**Figure 3:**
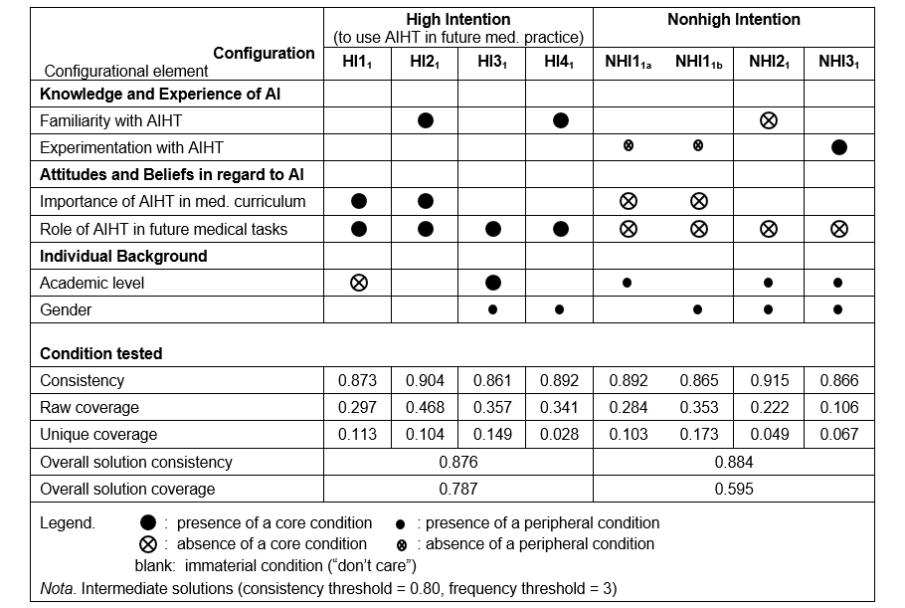
Configurations for the presence and absence of a high intention to use artificial intelligence (AI)–based health technologies (AIHTs) in future medical practice (*t*_1_). HI: high intention; med.: medical; NHI: nonhigh intention.

Similar to the results of the sufficiency analysis of the *t*_0_ data, results of the sufficiency analysis of the *t*_1_ data identify 4 intermediate solutions, that is, 4 causal configurations equally associated with a high intention to use AIHTs in future medical practice (*HI*1_1_, *HI*2_1_, *HI*3_1_, and *HI*4_1_). The 4 intermediate solutions derived from fsQCA are shown in Figure 3.

- The first high-intention configuration, *HI*1_1_, highlights the need for prospective physicians to have a strong belief in the role played by AIHTs in their future medical tasks (core condition) and to have positive attitudes toward the importance of AI in the medical curriculum (core condition). Furthermore, *HI*1_1_ is under the (core) condition that these individuals be in their first or second year of medical training.
- The second configuration,*HI*2_1_, also highlights the need for prospective physicians to have a strong belief in the role of AIHTs in their future medical tasks (core condition) and positive attitudes toward the importance of AI in the medical curriculum (core condition). However, *HI*2_1_ also includes a sufficient level of familiarity with AI technologies as a (core) condition for prospective physicians to have a strong intention to use AIHTs in their future practice, irrespective of their academic level and gender.
- The third configuration, *HI*3_1_, is the most parsimonious, in that it only includes (as a core condition) having a strong belief in the role to be played by AIHTs in supporting prospective physicians’ future medical tasks under the added condition of the physicians being in their third or later year of medical training (core condition) and being women (peripheral condition).
- The last configuration, *HI*4_1_, highlights the need to have a strong belief in the supporting role played by AIHTs in future medical tasks (core condition) and to have a high familiarity with AIHTs (core condition) under the (peripheral) condition that the prospective physicians be women.

At *t*_1_, there appear to be 4 different “causal recipes” for prospective physicians to develop a strong intention to use AIHTs in their future medical practice. Moreover, it is worth noting that, notwithstanding the prior analysis of necessary conditions, a strong belief in the role of AIHTs in support of future medical tasks appears to be a necessary condition because it is present in all 4 high-intention configurations [49].

#### Configurations for Nonhigh Intention to Use AIHTs in Future Medical Practice (*t*_1_)

Demonstrating causal asymmetry in a fashion similar to what was done for the *t*_0_ data and as presented in Figure 3, further results of the fsQCA analysis of the *t*_1_ data identify 4 causal configurations associated with nonhigh intention to use AIHTs in medical practice (*NHI*1_1_*_a_*, *NHI*1_1_*_b_*, *NHI*2_1_, and *NHI*3_1_). Note that the first 2 configurations share the same core conditions and thus may be considered as “second-order” solutions with regard to equifinality [22]. The absence of a strong belief in the role of AIHTs in support of future medical tasks is the core condition that is shared by all 4 configurations and is thus a condition that would be detrimental to the future use of AIHTs in prospective physicians’ medical practice.

### Comparative Analyses (*t*_0_ and *t*_1_)

A comparative look at Figures 2 and 3 allowed us to make the following observations regarding the high intention configurations identified in the replication study (peri–COVID-19 pandemic, *t*_1_; n=138), as compared with those identified in the initial study (pre–COVID-19 pandemic, *t*_0_; n=184):

- The *HI*1_1_ configuration is nearly identical to *HI*1_0_, as only the individual background conditions vary in importance (core vs peripheral condition).
- The *HI*2_1_ configuration substitutes the familiarity with AIHTs (core) condition for the experimentation with AIHTs (core) condition, that is, substitutes AI knowledge for AI experience when compared with *HI*2_0_.
- The *HI*3_1_ configuration is nearly identical to *HI*3_0_, as only the individual background conditions vary in importance (core vs peripheral condition).
- The *HI*4_1_ configuration includes the familiarity with AIHTs (core) condition and excludes the academic level (peripheral) condition when compared with *HI*3_0_.

With regard to the nonhigh intention configurations, differences between the configurations at *t*_0_ (Figure 2) and *t*_1_ (Figure 3) may be tentatively attributed to the significant differences between the 2 samples (Table S1 in Multimedia Appendix 2), that is, to the lesser familiarity and experimentation with AI of the students at *t*_1_ when compared with those at *t*_0_ and not to the differences in their individual background.

These observations are indicative of the robustness of our results and overall validity of the configurations that emerged from this study.

## Discussion

### Principal Findings

Our study first shows that a strong belief in the role of AIHTs in future medical tasks consistently figure as part of sufficient configurations and as the only individually necessary condition for future (intended) use of AI (Figures 2 and 3). This condition is also the only one that is causally symmetric, that is, the students who have a low intention to use AI are the students who do not believe AI will play an important role in their future profession. With regard to the other conditions, we uncover distinct AI profiles, that is, configurations, that describe equifinal sufficient solutions associated with the outcome of high intention toward AI. For the most prevalent profile of students in the early years of medical education, the core condition of a strong belief in the role of AI was sufficient, together with the condition that they have favorable attitudes toward the importance of AI (peripheral at *t*_0_ and core at *t*_1_). For the second major AI profile, which applies across academic levels and genders, a favorable attitude toward AI and a form of knowledge or experience with AI (experimentation in *t*_0_ and familiarity in *t*_1_) were conditions for the outcome of high behavioral intention. Finally, for a distinct profile of women participants with a high intention to use AI, a strong belief in the role of AI remained the only additional core condition (complemented by familiarity with AIHTs in the second sample). This last profile was mostly observed for students in their later years of medical education.

Beyond these nuanced findings, an additional fundamental insight is that being familiar with AI and having experimented with AI, considered individually, are not necessary conditions for students’ intention to use AI in their future practice. This was confirmed by both forms of analysis, the fsQCA and NCA. As Hanckel et al [39] noted, identifying such conditions that—against conventional expectations—are not individually necessary for the outcome can be seen as a key strength of fsQCA. With prior research and discourse primarily focusing on curriculum design and the teaching of AI competencies (ie, knowledge and familiarity), our findings show that these efforts are expected to be ineffective in shaping medical students’ behavioral intentions. Instead, the evidence from our study suggests that their belief regarding the role of AIHTs deserves more attention.

In interpreting the findings from this study, one should also appreciate the fsQCA method and its unique strengths. Originally applied to comparative policy analyses, that is, small sample size, noninterventional contexts involving complex causal relationships, QCA is increasingly valued in health care contexts [39]. The benefit of fsQCA, compared with traditional, regression-based approaches, is that it deals with profiles, or configurations of conditions, instead of assuming population homogeneity, independence of variables, and constant marginal effects. In our context, the fsQCA method was capable of capturing nuanced findings, including the findings that (1) the intention to use AIHTs is only observed when prospective physicians have a strong belief in the role of AI (individually necessary condition); (2) certain AI profiles, that is, combinations of knowledge and experience, attitudes and beliefs, and academic level and gender, are always associated with high intentions to adopt AI (equifinal and sufficient configurations); and (3) profiles associated with nonhigh intentions cannot be inferred from AI profiles associated with high intentions (causal asymmetry). Furthermore, the findings displayed in Figures 2 and 3 also indicate that the sufficient configurations depend on the academic level and gender, offering starting points for more targeted educational initiatives.

### Implications

A key implication for medical education is that the intention to adopt AI is observed only when students have a strong belief in the role of AI in medicine. Prior research offers suggestions of requisite AI-related skills and selections of corresponding curricular contents [52-55]. In our work, we emphasize that beyond teaching basic AI skills, the medical curriculum should also consider the roles of attitudes, beliefs, and behavioral intentions. To accomplish this, medical schools may foster an environment in which prospective physicians can explore, discuss, and develop their views with peers and expert practitioners early on. It would be fair to provide students with accurate information and access to experts to assist the formation of attitudes related to AIHTs and to facilitate the self-selection into medical specialties. In a nutshell, educational efforts should avoid producing students with AI-related skills but no intention of using AIHTs. Furthermore, we advise educators to adapt their teaching approaches to the different AI profiles, taking into consideration that students in the early years may want to appreciate the importance of AI in their future profession, whereas students in the later years may use AI when they have acquired enough knowledge. Ideally, educational initiatives should be adapted to the AI profiles related to AI attitudes and beliefs as well as AI-related familiarity and experimentation.

### Limitations

This exploratory study has a few limitations that can serve as a starting point for future research. First, the scope of our study was restricted to a single medical school in Canada, and our findings may not be generalizable to other medical education contexts, especially when the career paths of physicians, country’s development levels, health care systems, or regulations related to the medical profession differ. Second, although our sampling frame aimed to cover a broad variety of cases, several theoretical cases (ie, combinations of conditions) were not observed in the truth table. However, the highest number of cases corresponding to a single configuration do not reflect >10% of the data set, suggesting that the data set provides a strong empirical foundation for our findings [40]. Given that QCA, as an analytical method, is appropriate for small samples (eg, 10 to 30 cases), it is essential that there are no single configurations that represent large parts of the data set and to consider the logical remainder in the truth table when interpreting the results [40]. Third, the data collection instrument was created for this study and relies on the general terms such as AI, machine learning, and big data analytics. Future research could take this as a starting point to develop more specific operational definitions, not only of AI in the context of health care but also of AIHTs. Fourth, the survey is an observational and noninterventional data collection method. Further research is needed to ascertain the degree to which selected variables may change through intervention or the extent to which the efforts to inform medical students about the expected impacts of AI on their future practice enable them to self-select into the different specialties.

### Conclusions

The future of medical practice is expected to feature AI technologies, raising the question of how prospective physicians are best prepared for the new demands of the profession. Considerable work has been done related to the selection of AI topics and AIHT competencies for curriculum redesign. However, being competent in the use of AIHTs does not necessarily coincide with the behavioral intent to adopt these technologies. In this context, our work explains behavioral intent based on fsQCA, which identifies strong belief in the role of AIHTs as the only necessary condition, and dissociates different AI profiles as sufficient configurations. A replication showed that the findings remained stable, even after the advent of the COVID-19 pandemic. Going forward, these insights suggest that educators should go beyond teaching AIHT competencies and consider students’ beliefs and attitudes, which are intricately related to the intended adoption of AIHTs in their future practice.

## Appendix

Multimedia Appendix 1 Research variables’ measures.

Multimedia Appendix 2 Reliability and validity of research variables.

Multimedia Appendix 3 Details of the necessary condition analysis.

## Abbreviations

AI: artificial intelligence
AIHT: artificial intelligence–based health technology
CMB: common method bias
CMV: common method variance
fsQCA: fuzzy-set qualitative comparative analysis
NCA: necessary condition analysis
TAM: technology acceptance model

## Data Availability

Data and code for the analyses are available at GitHub and Zenodo. They can be accessed via the Zenodo website [56].
