# Supplementary material for: Understanding Prospective Physicians’ Intention to Use Artificial Intelligence in Their Future Medical Practice: Configurational Analysis
Source: JMIR Med Educ. 2023 Mar 22;9:e45631. doi: 10.2196/45631 (PMC10131981; doi:10.2196/45631)
Supplement: Multimedia Appendix 1 [file mededu_v9i1e45631_app1.docx]

## Appendix A: Research variables’ measures

**Familiarity with AIHT**

What is your level of familiarity with each of the following technologies and applications?

Very low Low Moderate High Very high

Artificial intelligence □ □ □ □ □

Machine learning □ □ □ □ □

Big data analytics □ □ □ □ □

**Experimentation with AIHT**

To what extent have you been exposed to each of the following technologies and applications during your medical training (courses and/or internships)?

Very low Low Moderate High Very high

Artificial intelligence □ □ □ □ □

Machine learning □ □ □ □ □

Big data analytics □ □ □ □ □

**Importance of AIHT in the medical curriculum**

To what extent do you agree or disagree with the fact that medical students should receive more training on the foundations, uses, challenges, risks and benefits associated to each of the following technologies and applications?

Totally Rather Neither disagree Rather Totally

disagree disagree /nor agree agree agree

Artificial intelligence □ □ □ □ □

Machine learning □ □ □ □ □

Big data analytics □ □ □ □ □

**Role of AIHT in future medical tasks**

In your opinion, to what extent will artificial intelligence and machine learning have an effect on each of the following aspects of medical practice?

Very negative Rather negative No Rather positive Very positive

effect effect effect effect effect

Prevention of illnesses □ □ □ □ □

Diagnosis of illnesses □ □ □ □ □

Treatment of illnesses □ □ □ □ □

Prognosis of illnesses □ □ □ □ □

Patient-physician relationship □ □ □ □ □

**Intention to use AIHT in future medical practice**

To what purposes do you intend to use AI-based technologies and applications in your medical practice at the end of your studies?

Yes No

To analyze images of a radiological nature □ □

To analyze images of a photographical nature (e.g., fundus of the eye) □ □

To analyze images of a pathological nature (e.g., biopsy specimen) □ □

To make diagnostics concerning my patients □ □

To make prognostics concerning my patients □ □

To determine my patients’ care protocols □ □

To analyze the data from my anamnesis in order to generate an opinion □ □

To supervise and evaluate my interviews with my patients □ □
