# Supplementary material for: Understanding Prospective Physicians’ Intention to Use Artificial Intelligence in Their Future Medical Practice: Configurational Analysis
Source: JMIR Med Educ. 2023 Mar 22;9:e45631. doi: 10.2196/45631 (PMC10131981; doi:10.2196/45631)
Supplement: Multimedia Appendix 2 [file mededu_v9i1e45631_app2.docx]

## Appendix 2: Reliability and validity of research variables

Table S1. Reliability, descriptive statistics, and comparison of research variables.

| **Research Construct**  Research variable | **t_0_** (n = 184) | | | | | **t_1_** (n = 138) | | | | | s |  |
| --- | --- | --- | --- | --- | --- | --- | --- | --- | --- | --- | --- | --- |
|  | α^a^ | mean | stdev | min | max | α^a^ | mean | stdev | min | max | **T**^b^ | *P* |
|  |  |  |  |  |  |  |  |  |  |  |  |  |
| **Knowledge and Experience of AI** |  |  |  |  |  |  |  |  |  |  |  |  |
| Familiarity with AIHT | 0.87 | 1.95 | 0.89 | 1.0 | 5.0 | 0.82 | 1.76 | 0.82 | 1.0 | 4.3 | 2.0* | .047 |
| Experimentation with AIHT | 0.66 | 1.25 | 0.45 | 1.0 | 4.0 | 0.73 | 1.16 | 0.39 | 1.0 | 3.7 | 1.9 | .052 |
| **Attitudes and Beliefs in regard to AI** |  |  |  |  |  |  |  |  |  |  |  |  |
| Importance of AIHT in the medical curric. | 0.84 | 3.52 | 0.76 | 1.0 | 5.0 | 0.84 | 3.50 | 0.71 | 1.0 | 5.0 | 0.3 | .801 |
| Role of AIHT in future medical tasks | 0.82 | 3.56 | 0.60 | 2.0 | 5.0 | 0.79 | 3.48 | 0.51 | 2.4 | 5.0 | 1.2 | .243 |
| **Individual Background** |  |  |  |  |  |  |  |  |  |  |  |  |
| Academic level^c^ |  | 0.59 |  | 0 | 1 |  | 0.57 |  | 0 | 1 | 0.4 | .697 |
| Gender^d^ |  | 0.65 |  | 0 | 1 |  | 0.69 |  | 0 | 1 | -0.9 | .357 |
| **Behavioral Intention with regard to AI** |  |  |  |  |  |  |  |  |  |  |  |  |
| Intention to use AIHT in future med. pract. |  | 3.93 | 3.07 | 0 | 8 |  | 3.64 | 3.22 | 0 | 8 | 0.8 | .412 |

*: *P*<.05

^a^ Cronbach’s alpha coefficient of reliability [inappropriate for index variables]

^b^ two-tailed t-test (comparison of the means)

^c^ [0 = 1^st^ or 2^nd^ year / 1 = 3^rd^, 4^th^ or 5^th^ year]

^d^ [0 = male / 1 = female]

Table S2. Convergent validity and intercorrelations of the research variables.

| **Research Construct**  Research variable | **AVE^a^** | | **Correlation matrix** | | | | | | | | | | | |
| --- | --- | --- | --- | --- | --- | --- | --- | --- | --- | --- | --- | --- | --- | --- |
|  | **t_0_** | **t_1_** | **t_0_**  (n = 184) | | | | | | **t_1_**  (n = 138) | | | | | |
|  |  |  | 1. | 2. | 3. | 4. | 5. | 6. | 1. | 2. | 3. | 4. | 5. | 6. |
| **Knowledge and Experience of AI** |  |  |  |  |  |  |  |  |  |  |  |  |  |  |
| 1. Familiarity with AIHT | .80 | .74 | - |  |  |  |  |  |  |  |  |  |  |  |
| 2. Experimentation with AIHT | .62 | .69 | .33 | - |  |  |  |  | .39 | - |  |  |  |  |
| **Attitudes and Beliefs in regard to AI** |  |  |  |  |  |  |  |  |  |  |  |  |  |  |
| 3. Importance of AIHT in the med. curric. | .76 | .77 | .39 | .08 | - |  |  |  | .20 | .10 | - |  |  |  |
| 4. Role of AIHT in future medical tasks | .64 | .51 | .42 | .17 | .48 | - |  |  | .18 | .06 | .39 | - |  |  |
| **Individual Background** |  |  |  |  |  |  |  |  |  |  |  |  |  |  |
| 5. Academic level | - | - | -.01 | .05 | -.18 | -.14 |  |  | -.13 | .00 | -.02 | -.15 | - |  |
| 6. Gender | - | - | -.42 | -.04 | -.22 | -.22 | -.06 | - | -.22 | .02 | -.00 | -.11 | .09 | - |
| **Behavioral Intention with regard to AI** |  |  |  |  |  |  |  |  |  |  |  |  |  |  |
| 7. Intention to use AIHT in future practice | - | - | .29 | .12 | .39 | .71 | -.04 | -.07 | .13 | .02 | .40 | .77 | -.03 | -.01 |

^a^average variance extracted [inappropriate for index variables]
