# Supplementary material for: Understanding Prospective Physicians’ Intention to Use Artificial Intelligence in Their Future Medical Practice: Configurational Analysis
Source: JMIR Med Educ. 2023 Mar 22;9:e45631. doi: 10.2196/45631 (PMC10131981; doi:10.2196/45631)
Supplement: Multimedia Appendix 3 [file mededu_v9i1e45631_app3.docx]

## Appendix C: Details of the necessary condition analysis (NCA)

Table C1. Results of necessary condition analysis (NCA) for high intention to use AIHT (t_0_).

| Configurational element | c-accuracy | ceiling  zone | scope | effect size | p-value |
| --- | --- | --- | --- | --- | --- |
| Familiarity with AIHT | 100% | 0.000 | 0.05 | 0.000 | 1.000 |
| Experimentation with AIHT | 100% | 0.000 | 0.96 | 0.000 | 1.000 |
| Importance of AIHT in the med. curric. | 100% | 0.000 | 0.96 | 0.000 | 1.000 |
| Role of AIHT in future med. tasks | 100% | 0.047 | 0.96 | 0.046 | 0.000 |
| Academic level | 100% | 0.000 | 0.96 | 0.000 | 1.000 |
| Gender | 100% | 0.000 | 0.96 | 0.000 | 1.000 |

*Nota*. CE-FDH ceiling technique

*Legend*. CE-FDH = ceiling envelopment with free disposal hull

Table C2. Results of NCA bottleneck level (%) for high intention to use AIHT (t_0_).

| Intention to use AIHT | Familiarity  with AIHT | Experimentation  with AIHT | Importance  of AIHT | Role  of AIHT | Academic  level | Gender |
| --- | --- | --- | --- | --- | --- | --- |
| 0 | NN | NN | NN | NN | NN | NN |
| 10 | NN | NN | NN | NN | NN | NN |
| 20 | NN | NN | NN | 1.0 | NN | NN |
| 30 | NN | NN | NN | 1.0 | NN | NN |
| 40 | NN | NN | NN | 1.0 | NN | NN |
| 50 | NN | NN | NN | 1.0 | NN | NN |
| 60 | NN | NN | NN | 1.0 | NN | NN |
| 70 | NN | NN | NN | 6.0 | NN | NN |
| 80 | NN | NN | NN | 6.0 | NN | NN |
| 90 | NN | NN | NN | 12.0 | NN | NN |
| 100 | NN | NN | NN | 58.0 | NN | NN |

*Nota*. CE-FDH ceiling technique

*Legend*. NN = not necessary CE-FDH = ceiling envelopment with free disposal hull

Table C3. Results of necessary condition analysis (NCA) for high intention to use AIHT (t_1_).

| Configurational element | c-accuracy | ceiling  zone | scope | effect size | p-value |
| --- | --- | --- | --- | --- | --- |
| Familiarity with AIHT | 100% | 0.000 | 0.89 | 0.000 | 1.000 |
| Experimentation with AIHT | 100% | 0.000 | 0.89 | 0.000 | 1.000 |
| Importance of AIHT in the med. curric. | 100% | 0.000 | 0.94 | 0.000 | 1.000 |
| Role of AIHT in future med. tasks | 100% | 0.028 | 0.94 | 0.030 | 0.021 |
| Academic level | 100% | 0.000 | 0.94 | 0.000 | 1.000 |
| Gender | 100% | 0.000 | 0.94 | 0.000 | 1.000 |

*Nota*. CE-FDH ceiling technique

*Legend*. CE-FDH = ceiling envelopment with free disposal hull

Table C4. Results of NCA bottleneck level (%) for high intention to use AIHT (t_1_).

| Intention  to use AIHT | Familiarity  with AIHT | Experimentation  with AIHT | Importance  of AIHT | Role  of AIHT | Academic  level | Gender |
| --- | --- | --- | --- | --- | --- | --- |
| 0 | NN | NN | NN | NN | NN | NN |
| 10 | NN | NN | NN | NN | NN | NN |
| 20 | NN | NN | NN | NN | NN | NN |
| 30 | NN | NN | NN | 2.0 | NN | NN |
| 40 | NN | NN | NN | 2.0 | NN | NN |
| 50 | NN | NN | NN | 2.0 | NN | NN |
| 60 | NN | NN | NN | 2.0 | NN | NN |
| 70 | NN | NN | NN | 2.0 | NN | NN |
| 80 | NN | NN | NN | 2.0 | NN | NN |
| 90 | NN | NN | NN | 2.0 | NN | NN |
| 100 | NN | NN | NN | 59.0 | NN | NN |

*Nota*. CE-FDH ceiling technique

*Legend*. NN = not necessary CE-FDH = ceiling envelopment with free disposal hull
